# The association between Korean workers’ presenteeism and psychosocial factors within workplaces

**DOI:** 10.1186/s40557-016-0124-1

**Published:** 2016-09-07

**Authors:** Yun-Sik Cho, Jae Bum Park, Kyung-Jong Lee, Kyoung-Bok Min, Chul-In Baek

**Affiliations:** 1 Department of Occupational and Environmental Medicine, Ajou University Hospital, Suwon, South Korea; 2 Department of Occupational and Environmental Medicine, Ajou University School of Medicine, Suwon, South Korea; 3 Department of Preventive Medicine, College of Medicine, Seoul National University, Seoul, South Korea

**Keywords:** Presenteeism, Workplace psychosocial factors, Korean Working Conditions Survey, Discrimination, Harassment, Job stress

## Abstract

**Background:**

Presenteeism, a concept that has recently undergone active study, is the act of attending work while sick. This study investigates the association between presenteeism and various psychosocial factors within workplaces.

**Methods:**

This study analyzed 29246 wage earners from the third Korean Working Conditions Survey (KWCS, 2011) data using the logistic regression analysis to investigate the association between presenteeism and various psychosocial factors within workplaces.

**Results:**

Among the 29246 wage earners, 6347 (21.7 %) showed presenteeism. Those who experienced age discrimination at work (adjusted odds ratio (aOR) 1.77: 95 % CI 1.56–2.00), educational background discrimination (aOR 1.35: 95 % CI 1.22–1.51), regional discrimination (aOR 1.55: 95 % CI 1.31–1.83), sexual discrimination (aOR 1.65: 95 % CI 1.41–1.94), employment type discrimination (aOR 2.13: 95 % CI 1.89–2.40), physical violence (aOR 1.92: 95 % CI 1.45–2.55), sexual harassment (aOR 2.90: 95 % CI 2.01–4.19), job insecurity (aOR 1.36: 95 % CI 1.18–1.56), work–life imbalance (aOR 1.38: 95 % CI 1.29–1.47), low job satisfaction (aOR 2.04: 95 % CI 1.91–2.17), no colleague support (aOR 1.11: 95 % CI 1.02–1.21), job stress (aOR 1.89: 95 % CI 1.76–2.02), emotional labor (aOR 1.50: 95 % CI 1.41–1.60), high work intensity (aOR 1.31: 95 % CI 1.23–1.38), and 3 groups of job strain that are passive group (aOR 1.09: 95 % CI 1.00–1.18), active group (aOR 1.39: 95 % CI 1.28–1.51), and high strain group (aOR 1.35: 95 % CI 1.24–1.46) showed an increased risk of presenteeism compared to their respective counterparts (*p* < 0.01).

**Conclusions:**

The study results confirmed the association between presenteeism and various psychosocial factors within workplaces. Considering that presenteeism negatively affects productivity and the mental and physical health of individuals, managing various psychosocial factors within workplaces is proposed to reduce presenteeism.

## 국문요약

**목표:**

프리젠티즘은 최근 활발히 연구되고 있는 개념으로 건강하지 않은 상태로 출근하여 일하는 것을 의미한다. 본 연구는 프리젠티즘과 다양한 직장 내 사회 심리적 요인들의 관계를 파악하기 위하여 수행되었다.

**방법:**

이 연구에서는 2011 년 실시된 제 3 차 근로환경조사 자료를 이용하여 임금 근로자 29,246 명을 대상으로 분석을 실시하였다. 프리젠티즘과 직장 내 사회 심리적 요인의 관계를 알아보기 위해 로지스틱 회귀분석을 사용하였다.

**결과:**

29,246 명의 임금근로자 중 프리젠티즘이 있는 근로자는 6,347 명 (21.7 %), 프리젠티즘이 없는 근로자는 22,899 명 (78.3 %) 이었다. 개인적 특성과 직업적 요인들을 보정하여 분석한 결과 프리젠티즘이 있는 경우가 없는 경우에 비해 연령차별이 있는 경우 1.77 배 (95 % CI 1.56-2.00), 학벌차별이 있는 경우 1.35 배 (95 % CI 1.22-1.51), 출신지역차별이 있는 경우 1.55 배 (95 % CI 1.31-1.83), 성차별이 있는 경우 1.65 배 (95 % CI 1.41-1.94), 고용형태차별이 있는 경우 2.13 배 (95 % CI 1.89-2.40), 신체적 폭력이 있는 경우 1.92 배 (95 % CI 1.45-2.55), 성희롱이 있는 경우 2.90 배 (95 % CI 2.01-4.19), 직무 불안정이 있는 경우 1.36 배 (95 % CI 1.18-1.56), 일-생활 균형이 맞지 않는 경우 1.38 배 (95 % CI 1.29-1.47), 직무 만족도가 낮은 경우 2.04 배 (95 % CI 1.91-2.17), 동료의 지지를 받지 못하는 경우 1.11 배 (95 % CI 1.02-1.21), 업무 스트레스를 받는 경우 1.89 배 (95 % CI 1.76-2.02), 감정 노동의 경우 1.50 배 (95 % CI 1.41-1.60), 직무 요구도가 높은 경우 1.31 배 (95 % CI 1.23-1.38), 직무 긴장도는 낮은 긴장도 그룹을 기준으로 수동적 그룹이 1.09 배 (95 % CI 1.00-1.18), 능동적 그룹이 1.39 배 (95 % CI 1.28-1.51), 높은 긴장도 그룹이 1.35 배 (95 % CI 1.24-1.46) 높게 나타났다.

**결론:**

본 연구 결과, 프리젠티즘과 직장 내 사회 심리적 요인들의 연관성을 확인할 수 있었다. 프리젠티즘이 생산성 및 개인의 정신적, 육체적 건강에 큰 영향을 미치는 만큼, 프리젠티즘을 줄이기 위한 여러 가지 방안 중 하나로 직장 내 사회 심리적 요인의 관리를 제시할 수 있을 것이다.

## Background

Presenteeism, a concept often perceived as the opposite of absenteeism, has been actively studied recently, and various definitions have been created. Kivimäki et al. [1] define presenteeism as feeling sick but attending work. Aronsson et al. [2] and Dew et al. [3] define it as attending work while ill, and Evans et al. [4] and Johansson et al. [5] define it as attending work despite feeling sick or being at work while being under conditions that would generally require absenteeism. Turpin et al. [6] have mentioned presenteeism as the decrease in productivity at work resulting from health problems, and Hemp defines presenteeism as the worker’s state of not fully functioning because of diseases or other medical reasons [7]. This study defines presenteeism as attending work while ill [8].

Many studies have recently reported that presenteeism decreases productivity at work [9–12]. In particular, Hemp states that the invisible cost resulting from presenteeism is much greater than other health-related costs [7], an assertion supported by the calculation of Stewart et al. [13] using 29000 US workers as participants; the lost productive time (LPT) cost resulting from presenteeism is over $150000 million, and the Health-and-Productivity Toolkit (2006) calculated the mean annual consumption cost for diseases per worker resulting from presenteeism to be $155.92 [14]. Regarding studies on presenteeism and the health association, studies have found that workers with no absence from work for 3 years showed coronary artery disease rates twice as high as those who had been absent among workers with cardiovascular disease risks [1]. Presenteeism has also been found to be a risk factor for future sick leave occurrences [15]. Furthermore, considering the study result that workers who take sick leaves experience 28 % fewer nonfatal injuries than those who do not take sick leaves [16], presenteeism is closely associated with productivity, work health, and work injury.

Many studies have investigated the association between presenteeism and various psychosocial factors within workplaces domestically and internationally. Ryu et al. [17], Jung et al. [18] and Gun et al. [19] report that job and psychosocial stresses affect presenteeism and Dew et al. [3] report that presenteeism increases in proportion with increases in job risks or physical workload. Aronsson et al. [2] report that presenteeism is found at a high rate among workers in the fields of treatment, well-being, and education services and that these tendencies are particularly evident when it is difficult to find substitute workers. In looking at the job design aspect, presenteeism is found at a high rate in cases of high work intensity [20], low work-speed control ability [21], heavy workload [2, 21], and low job satisfaction [22, 23]. Furthermore, presenteeism is found at a high rate in cases of time pressure [21, 24], low colleagues’ support [24], workaholism [25], and emotional or physical health symptoms [26, 27].

However, previous studies have focused primarily on work productivity, comparing presenteeism with absenteeism; many of these studies focus on small-scale industries; the related psychosocial factors are limited, presumably because the concept of presenteeism initially focused on worker productivity and economy, which cannot be explained with absenteeism alone. In addition, the study period has been relatively short. The same is true domestically, and the studies on presenteeism have been conducted within a few limited professions and factors. Thus, this study investigated presenteeism and its association with various psychosocial factors within workplaces using data from the third Korean Working Conditions Survey (KWCS, 2011). Among the psychosocial factors seen within workplaces, 18 items were analyzed, including stress, job intensity, job autonomy, job satisfaction, colleague support, and job insecurity with a focus on each factor’s association with presenteeism, the related sociodemographic variable, and work environments.

## Methods

### Study population

This study was conducted on workers aged 15 years old and older in 16 cities and provinces (including Jeju Island) as participants using the secondary data of the third KWCS. The survey was conducted in a one-on-one interview format with professional interviewers meeting with a total of 50032 respondents categorized by their work positions, e.g., employer/business owner with hired employees, self-employed business owners with no hired employees, wage earners, unpaid family workers and others (special workers). “Wage earner” was defined as one who works for a wage earner, individual, household, or business entity through an explicit or implicit work contract and is paid by wage, salary, daily payment, or goods. The final participants (29246) for this study were selected from those who worked more than a year among the 34788 wage earners left out of 50032 people surveyed by excluding the self-employed (8616), business owners (3098), unpaid family workers (2562), and others (968).

### Participants

For precise analysis of the association between presenteeism and various psychosocial factors within workplaces, psychosocial factors were selected from the previous studies using individual and job factors as controlling variables [28, 29].

Presenteeism was assigned to a participant when he or she answered “yes” to the survey question, “Have you ever worked in the past 12 months despite being sick?”

Among the psychosocial factors within workplaces, discrimination was determined in accordance with the answers to the question, “Have you ever experienced discrimination at work described in the following in the past 12 months?” Experience of age discrimination, educational background discrimination, regional discrimination, sexual discrimination, and employment contract discrimination were given as discrimination items. Answering “yes” to having been discriminated against in any of the aforementioned ways was counted as experiencing discrimination, and answering “no” indicated no discrimination. Regarding bullying and harassment, the items provided for the question, “Have you ever experienced at work the misfortunes described in the following in the past 12 months?” were physical violence, ostracizing/bullying, harassment, and sexual harassment. Answering “yes” was counted as experiencing bullying or harassment, and answering “no” indicated that there was no discrimination. Regarding job insecurity, answering “yes” to the statement, “I will lose this job within 6 months from now,” was determined to indicate the presence of job insecurity, while an answer of “no” marked an absence of job insecurity. Regarding work–life balance, answering “very reasonable” or “reasonable” to the question, “Do you think your work hours are adequate/reasonable for your home life and community activities?” was determined to indicate good work–life balance, and answering “very inadequate” or “not reasonable” was determined to indicate poor work–life balance. Regarding job satisfaction, answering “very much satisfied” or “satisfied” to the question, “What do you think about your work environment in general?” was determined to indicate high job satisfaction, and answering “not so much satisfied” or “not at all satisfied” was determined to indicate low job satisfaction. Regarding colleague and supervisor support, answering “mostly so” and “sometimes so” to the statements, “My colleagues help and support me” and “My supervisors help and support me” was determined to indicate high support, and answering “not so much so” or “not at all so” was determined to indicate low support. Regarding job stress, answering “always so”, “mostly so” or “sometimes so” to the statement, “I am under stress at work” was determined to indicate high stress, and answering “not so much so” or “not at all so” was determined to indicate low stress. Regarding emotional labor, answering “always so”, “mostly so” or “sometimes so” to the statement, “I have to hide my emotions at work” was counted as responding “yes” and answering “not so much so” or “not at all so” was counted as responding “no”. Participants assigned scores of 100 (all work hours), 90 (most of the work hours), 75 (three quarters of the work hours), 50 (half of the work hours), 25 (a quarter of the work hours), 10 (almost none), and 0 (none) to each of the seven answers in sub-items (A) “work at a high speed” and (B) “work with strict deadlines” under the question, “How much of the following circumstances are included in your work?” Afterwards, work intensity was determined to be low when the sum of the two items fell in the range of 0–35 and high when it fell in the range of 36–200. The Cronbach's alpha value, an internal consistency index of the tools, was 0.84 in this study for work intensity. Regarding job autonomy, five answer options were assigned for three statements: “My opinion is reflected when selecting work colleagues”, “I can rest at my convenience” and “I can influence major decision-making at work”. Five scores—i.e., 1 (always so), 2 (mostly so), 3 (sometimes so), 4 (not much so), 5 (not at all so)—were assigned for each statement, and the sums were then divided by 3. The obtained value was compared with the median value, 3.33, to be determined as “high job autonomy” when lower than the mean value and “low job autonomy” when higher than the mean value. The Cronbach's alpha value for job autonomy was 0.66. Regarding job strain, participants were assigned to the low strain group when work intensity was low and job autonomy was high, the passive group when work intensity and job autonomy were low, the active group when work intensity and job autonomy were high, and the high strain group when work intensity was high and job autonomy was low [30]. The overview report (2012) of the Fifth European Working Conditions Survey was referred to for these item answers [31].

Individual factors included sex, age, smoking behavior, drinking, education, and monthly income. Participants within these groups were then categorized by sex as either male or female and by age into one of three groups: 15–29, 30–49 and 50 and above. They were also categorized by smoking behavior into the three groups of “never smoked”, “quit smoking” and “currently smoking” as well as by drinking habits (“do not drink”, “drink less than once a week” and “drink more than twice a week”). Participants were categorized into one of three groups by their education level: pre-middle school graduates, high school graduates, and college and post-graduates. The groups were then further categorized by monthly income level into one of four groups: 1ess than 1 million won, greater than 1 million won and less than 2 million won, greater than 2 million won and less than 3 million won, and over 3 million won. As for professional factors, participants were categorized as being either full-time or part-time workers; as having non-shift or shift schedules by work schedule; and as working less than 35 h weekly, 35–44 h weekly or over 45 h weekly. As for job types, participants were categorized into the groups of professional (professional technicians, senior management positions), office, service (sales, service) and manual (skilled, semi-skilled, non-skilled, agriculture, and forestry).

### Data analysis

Sociodemographic and job types were analyzed, and then the chi-square method was used to investigate the individual association with presenteeism. After related factors were modified, logistic regression analysis was conducted to investigate the association between presenteeism and psychosocial factors within workplaces, and the odds ratio was analyzed accordingly. All results were analyzed by applying the weight. SPSS (Statistical Package for the Social Science) for Windows version 19.0 (SPSS INC: Chicago, IL, USA; 2010) was used for all analyses.

## Results

### General participant characteristics

There were 29246 participants in total, and their characteristics were analyzed for each factor using frequency analysis. There were 17812 male (60.9 %) and 11434 female (39.1 %) participants. Three groups were categorized by age range, including 5101 participants (7.4 %) in the range of 15 to 29 years, 17272 (59.1 %) in the range of 30 to 49 years, and 6873 (23.5 %) in the range of 50 years and above. The three groups categorized by smoking behavior included 15639 participants (53.5 %) categorized as “never” 3230 (11.0 %) categorized as “quit” and 10377 (35.5 %) categorized as “current”. The three groups categorized by drinking habits included 6184 participants (21.1 %) categorized as “do not drink”, 15050 (51.5 %) categorized as “drink less than once a week” and 8012 (27.4 %) categorized as “drink more than twice a week”. Three groups were categorized by education: 2750 participants (9.4 %) were categorized as “pre-middle school graduates,” 10193 (34.9 %) as “high school graduates,” and 16304 (55.7 %) as “college and post-graduates”. Four groups were categorized by monthly income level: 2339 (8.0 %) as “1ess than 1 million won”, 10909 (37.3 %) as “greater than 1 million won and less than 2 million won”, 9113 (31.2 %) as “greater than 2 million won and less than 3 million won” and 6880 (23.5 %) as “greater than 3 million won”. In looking at professional factors, four groups were categorized by job types: 2541 (8.7 %) as “professional”, 9467 (32.4 %) as “office”, 7426 (25.4 %) as “service” and 9813 (33.6 %) as “manual”. There were relatively greater numbers in the areas of office work and manual labor. Two groups were categorized by employment contract: 27683 (94.7 %) as “full-time” and 1563 (5.3 %) as “part time”. Two groups were categorized by work schedule: 2799 (9.6 %) as “non-shift” and 26448 (90.4 %) as “shift”. Three groups were categorized by weekly work hours: 1557 (5.3 %) as “less than 35 h”, 11133 (38.1 %) as “35–44 h” and 16557 (55.6 %) as “over 45 h” (Table 1).

**Table 1:** The sociodemographic characteristics of the study population

Characteristics		N	%
Sex	Male	17,812	60.9
	Female	11,434	39.1
Age (years)	15–29	5,101	17.4
	30–49	17,272	59.1
	≥50	6,873	23.5
Smoking	Never	15,639	53.5
	Quit	3,230	11.0
	Current	10,377	35.5
Drinking	No	6,184	21.1
	≤1/week	15,050	51.5
	≥2/week	8,012	27.4
Education	Below Middle School	2,750	9.4
	High School	10,193	34.9
	College and beyond	16,304	55.7
Income (10,000won/month)	<100	2,339	8.0
	100–199	10,909	37.3
	200–299	9,113	31.2
	≥300	6,880	23.5
Job type	Professional	2,541	8.7
	Office	9,467	32.4
	Service	7,426	25.4
	Manual	9,813	33.6
Employment contract	Full time	27,683	94.7
	Part time	1,563	5.3
Work schedule	Nonshift	26,448	90.4
	Shift	2,799	9.6
Working hours/week	<35	1,557	5.3
	35–45	11,133	38.1
	≥45	16,557	56.6

### Existence of presenteeism based on Participants’ sociodemographic characteristics and occupational factors

As for presence of presenteeism, 6347 participants (21.7 %) exhibited presenteeism, and 22899 participants (78.3 %) did not. The differences in each factor’s influence on the presence of presenteeism were analyzed using the chi-square method, and significant differences existed between the factors’ associations with presenteeism. In the case of sex, more female participants were found in the group exhibiting presenteeism (2809 female participants, 24.6 %) than were found in the group without presenteeism, while in the case of age, the highest rate of presenteeism was found in the group of individuals aged 50 and above (1552 participants, 22.6 %). In the case of smoking behavior, the group exhibiting presenteeism showed a higher rate of “quit” cases (757 participants, 23.4 %) than did the group without presenteeism, while in the case of drinking habits, the higher rate (1785 participants, 22.3 %) was found in the “more than twice a week” group; this difference was statistically significant. In the case of education, the group exhibiting presenteeism showed a higher rate of “below middle-school graduates” (750 participants, 27.3 %) than the group without presenteeism, and in the case of mean monthly income, the group with the income level of “greater than 1 million and less than 2 million” (2573 participants, 23.6 %) showed a higher rate than others. In the case of job type, the group exhibiting presenteeism showed the highest rate in the “office” group (607 participants, 23.9 %), higher than the group without presenteeism, while in the case of employment contract, the full-time group (6081 participants, 22.0 %) showed a high ratio. As for work schedule, the group exhibiting presenteeism showed a higher rate in the “shift” group (716 participants, 25.6 %), while in the case of weekly work hours, cases of “over 45 h” (3976 participants, 24.0 %) showed a significantly high ratio (Table 2).

**Table 2:** Comparisons of the characteristics of the study subjects according to the presenteeism

Characteristics		Presenteeism n (%)		*P*-value
		Yes	No	
		6,347 (21.7)	22,899 (78.3)	
Sex	Male	3,538 (19.9)	14,274 (80.1)	<0.001
	Female	2,809 (24.6)	8,625 (75.4)	
Age (years)	15–29	1,000 (19.6)	4,101 (80.4)	<0.001
	30–49	3,795 (22.0)	13,477 (78.0)	
	≥50	1,552 (22.6)	5,320 (77.4)	
Smoking	Never	3,423 (21.9)	12,215 (78.1)	0.006
	Quit	757 (23.4)	2,473 (76.6)	
	Current	2,167 (20.9)	8,211 (79.1)	
Drinking	No	1,275 (20.6)	4,909 (79.4)	0.049
	≤1/week	3,288 (21.8)	11,762 (78.2)	
	≥2/week	1,785 (22.3)	6,227 (77.7)	
Education	-Middle School	750 (27.3)	2,000 (72.7)	<0.001
	High School	2,254 (22.1)	7,939 (77.9)	
	College-	3,343 (20.5)	12,960 (79.5)	
Income (10,000won/month)	<100	524 (22.4)	1,816 (77.6)	<0.001
	100–199	2,573 (23.6)	8,335 (76.4)	
	200–299	1,804 (19.8)	7,309 (80.2)	
	≥300	1,444 (21.0)	5,436 (79.0)	
Job type	Professional	1,975 (20.9)	7,492 (79.1)	0.009
	Office	607 (23.9)	1,934 (76.1)	
	Service	1,610 (21.7)	5,815 (78.3)	
	Manual	2,155 (22.0)	7,658 (78.0)	
Employment contract	Full time	6,081 (22.0)	21,603 (78.0)	<0.001
	Part time	267 (17.1)	1,296 (82.9)	
Work schedule	Nonshift	5,632 (21.3)	20,816 (78.7)	<0.001
	Shift	716 (25.6)	2,083 (74.4)	
Working hours/week	<35	277 (17.8)	1,279 (82.2)	<0.001
	35–45	2,103 (18.9)	9,030 (81.1)	
	≥45	3,967 (24.0)	12,590 (76.0)	

### Presenteeism and its association with psychosocial factors within workplaces

To investigate presenteeism and its association with psychosocial factors within workplaces, the chi-square and binomial logistic regression analysis was performed. The chi-square analysis showed that most of psychosocial factors has significant correlation with presenteeism except some factors like sexual harassment and job autonomy for men and bullying, harassment and job insecurity for women (Table 3). The odds ratio and 95 % conference interval were calculated using binomial logistic regression analysis. First, presenteeism and its association with psychosocial factors within workplaces were analyzed without controlling variables; then, sex, age, smoking behavior, drinking habits, education, and monthly mean income were revised in Model A, and additional items of job types, employment contract, work schedule, and weekly work hours were revised in Model B. As a result, in looking at presenteeism and its association with psychosocial factors within workplaces, presenteeism appeared 1.77 more times (95 % CI 1.56–2.00) among those receiving experience of age discrimination than it did among those who did not, 1.35 more times (95 % CI 1.22–1.51) among those receiving education discrimination than it did among those who did not, 1.55 more times (95 % CI 1.31–1.83) in cases of regional discrimination than in those without, 1.65 more times (95 % CI 1.41–1.94) in cases of sexual discrimination than in those without, 2.13 more times (95 % CI 1.89–2.40) in cases of contract discrimination than in those without, 1.92 more times (95 % CI 1.45–2.55) in cases of physical violence than in those without, and 2.90 more times (95 % CI 2.01–4.19) in cases of sexual harassment than in those without, demonstrating statistically significant increase in the risks. However, in the case of ostracizing and bullying harassment, statistical significance was not observed (*p* = 0.073). Furthermore, cases of job insecurity showed presenteeism 1.36 times (95 % CI 1.18–1.56) more than cases without, cases of work-life imbalance showed presenteeism 1.38 times (95 % CI 1.29–1.47) more than cases without, and cases of low job satisfaction showed presenteeism 2.04 times (95 % CI 1.91–2.17) more than cases without, demonstrating statistically significant increase in the risks. Similarly, cases of absence of colleague support showed presenteeism 1.11 times (95 % CI 1.02–1.21) more than cases without, cases of work stress showed presenteeism 1.89 times (95 % CI 1.76–2.02) more than cases without, and cases of emotional labor and hiding one’s emotions showed presenteeism 1.50 times (95 % CI 1.41–1.60) more than cases without, demonstrating an increase in the risks. However, the presence of supervisor support did not show statistically significant value (*p* = 0.378). Cases of high work intensity showed presenteeism 1.31 times (95 % CI 1.23–1.38) more than cases of low work intensity, whereas job autonomy did not show much association (*p* = 0.342). In the case of job strain, the passive group showed presenteeism 1.09 times (95 % CI 1.00–1.18) higher than the low-strain group, while the active and high-strain groups showed presenteeism 1.39 (95 % CI 1.28–1.51) and 1.35 times (95 % CI 1.24–1.46) higher than the low-strain group, respectively, demonstrating a statistically significant increase in the risks (Table 4).

**Table 3:** Comparison of presenteeism on workplace psychosocial factors stratified with gender

Characteristics		Presenteeism n (%)					
		Male			Female		
		Yes	No	*P*-value	Yes	No	*P*-value
Experience of age discrimination	no	3,305 (19.4)	13,766 (80.6)	<0.001	2,626 (24.0)	8,293 (76.0)	<0.001
	yes	234 (31.5)	508 (68.5)		183 (35.5)	332 (64.5)	
Educational background discrimination	no	3,261 (19.5)	13,460 (80.5)	<0.001	2,579 (24.2)	8,085 (75.8)	<0.001
	yes	277 (25.4)	814 (74.6)		230 (29.9)	540 (70.1)	
Regional discriminatio	no	3,417 (19.6)	13,995 (80.4)	<0.001	2,726 (24.4)	8,439 (75.6)	<0.001
	yes	121 (30.3)	279 (69.8)		83 (30.9)	186 (69.1)	
Sexual discrimination	no	3,451 (19.7)	14,024 (80.3)	0.003	2,663 (24.1)	8,385 (75.9)	<0.001
	yes	88 (26.0)	250 (74.0)		146 (37.8)	240 (62.2)	
Employment type discrimination	no	3,288 (19.3)	13,772 (80.7)	<0.001	2,597 (23.8)	8,332 (76.2)	<0.001
	yes	251 (33.3)	502 (66.7)		212 (42.0)	293 (58.0)	
Physical violence	no	3,490 (19.7)	14,182 (80.3)	<0.001	2,778 (24.4)	8,584 (75.6)	<0.001
	yes	48 (34.3)	92 (65.7)		31 (43.7)	40 (56.3)	
Bullying, harassment	no	3,520 (19.8)	14,234 (80.2)	0.017	2,798 (24.6)	8,597 (75.4)	0.356
	yes	19 (32.2)	40 (67.8)		11 (28.2)	28 (71.8)	
Sexual harassment	no	3,529 (19.8)	14,253 (80.2)	0.072	2,764 (24.4)	8,584 (75.6)	<0.001
	yes	10 (32.3)	21 (67.7)		45 (52.3)	41 (47.7)	
Job insecurity	no	3,356 (19.6)	13,770 (80.4)	<0.001	2,695 (24.5)	8,309 (75.5)	0.185
	yes	182 (26.5)	504 (73.5)		114 (26.5)	316 (73.5)	
Work-life balance	good	2,282 (18.0)	10,417 (82.0)	<0.001	1,989 (22.7)	6,787 (77.3)	<0.001
	poor	1,257 (24.6)	3,857 (75.4)		820 (30.9)	1,838 (69.1)	
Job satisfaction	high	2,100 (16.3)	10,809 (83.7)	<0.001	1,904 (21.7)	6,886 (78.3)	<0.001
	low	1,438 (29.3)	3,465 (70.7)		905 (34.2)	1,739 (65.8)	
Colleagues support	high	2,934 (19.3)	12,276 (80.7)	<0.001	2,425 (25.5)	7,103 (74.5)	0.005
	low	537 (25.6)	1,563 (74.4)		287 (22.1)	1,011 (77.9)	
Supervisors support	high	2,837 (19.3)	11,882 (80.7)	<0.001	2,349 (25.2)	6,957 (74.8)	0.004
	low	660 (23.0)	2,211 (77.0)		423 (22.4)	1,469 (77.6)	
Job stress	low	597 (12.5)	4,165 (87.5)	<0.001	604 (17.8)	2,788 (82.2)	<0.001
	high	2,941 (22.5)	10,109 (77.5)		2,205 (27.4)	5,837 (72.6)	
Emotional labor	no	964 (14.8)	5,530 (85.2)	<0.001	817 (21.4)	2,992 (78.6)	<0.001
	yes	2,574 (22.7)	8,744 (77.3)		1,992 (26.1)	5,633 (73.9)	
Work intensity	low	1,294 (16.2)	6,670 (83.8)	<0.001	1,241 (22.5)	4,269 (77.5)	<0.001
	high	2,245 (22.8)	7,604 (77.2)		1,568 (26.5)	4,356 (73.5)	
Work autonomy	high	1,721 (19.8)	6,961 (80.2)	0.455	1,150 (23.5)	3,749 (76.5)	0.010
	low	1,817 (19.9)	,7313 (80.1)		1,659 (25.4)	4,876 (74.6)	
Job strain	Low strain	828 (16.5)	4,189 (83.5)	<0.001	640 (22.3)	2,225 (77.7)	<0.001
	Passive group	865 (17.9)	3,972 (82.1)		920 (24.1)	2,892 (75.9)	
	Active group	893 (24.4)	2,772 (75.6)		510 (25.1)	1,524 (74.9)	
	High strain	952 (22.2)	3,341 (77.8)		739 (27.1)	1,984 (72.9)

**Table 4:** Odds ratios of presenteeism on workplace psychosocial factors from logistic regression models

		Crude OR (95 % CI)	Model A OR (95 % CI)	Model B OR (95 % CI)
Experience of age discrimination	no	1	1	1
	yes	1.84 (1.63–2.08)	1.77 (1.56–2.00)	1.77 (1.56–2.00)
Educational background discrimination	no	1	1	1
	yes	1.38 (1.24–1.54)	1.38 (1.24–1.54)	1.35 (1.22–1.51)
Regional discrimination	no	1	1	1
	yes	1.60 (1.36–1.90)	1.58 (1.34–1.87)	1.55 (1.31–1.83)
Sexual discrimination	no	1	1	1
	yes	1.75 (1.49–2.05)	1.70 (1.45–1.99)	1.65 (1.41–1.94)
Employment type discrmination	no	1	1	1
	yes	2.19 (1.94–2.46)	2.15 (1.91–2.42)	2.13 (1.89–2.40)
Physical violence	no	1	1	1
	yes	2.18 (1.65–2.88)	2.06 (1.55–2.73)	1.92 (1.45–2.55)
Bullying, harassment	no	1	1	1
	yes	1.61 (1.05–2.47)	1.55 (1.00–2.39)	1.49 (0.96–2.31)
Sexual harassment	no	1	1	1
	yes	3.16 (2.20–4.55)	2.89 (2.01–4.18)	2.90 (2.01–4.19)
Job insecurit	no	1	1	1
	yes	1.32 (1.15–1.51)	1.30 (1.13–1.49)	1.36 (1.18–1.56)
Work–life balance	good	1	1	1
	poor	1.47 (1.38–1.56)	1.46 (1.38–1.56)	1.38 (1.29–1.47)
Job satisfaction	high	1	1	1
	low	1.99 (1.88–2.11)	2.04 (1.91–2.17)	2.04 (1.91–2.17)
Colleagues support	high	1	1	1
	low	1.16 (1.06–1.26)	1.13 (1.03–1.23)	1.11 (1.02–1.21)
Supervisors support	high	1	1	1
	low	1.07 (1.00–1.15)	1.03 (0.95–1.11)	1.04 (0.96–1.12)
Job stress	low	1	1	1
	high	1.87 (1.74–2.00)	1.94 (1.81–2.09)	1.89 (1.76–2.02)
Emotional labor	no	1	1	1
	yes	1.52 (1.43–1.61)	1.55 (1.45–1.64)	1.50 (1.41–1.60)
Work intensity	low	1	1	
	high	1.31 (1.24–1.39)	1.31 (1.23–1.38)	1.31 (1.23–1.38)
Work autonomy	high	1	1	1
	low	1.06 (1.01–1.13)	1.02 (0.97–1.09)	1.03 (0.97–1.09)
Job strain	Low strain	1	1	1
	Passive group	1.14 (1.05–1.23)	1.10 (1.01–1.19)	1.09 (1.00–1.18)
	Active group	1.43 (1.31–1.55)	1.42 (1.30–1.54)	1.39 (1.28–1.51)
	High strain	1.39 (1.28–1.50)	1.33 (1.23–1.45)	1.35 (1.24–1.46)

Model A: Adjusted for sex, age group, smoking, drinking, education, income

Model B: Model A + Adjusted for job type, employment contract, work schedule, working hours

*OR* = odds ratio, *CI* = confidence interval

## Discussion

To investigate presenteeism and its association with psychosocial factors within workplaces, this study investigated presenteeism—with wage earners as participants—and its association with participants’ sociodemographic characteristics and job types. The study analyzed 18 psychosocial factors within workplaces to determine each factor’s effect on presenteeism.

Analysis of the participants’ sociodemographic characteristics showed that presenteeism has significant correlation with sex, age, smoking behavior, drinking habits, education, and mean monthly income, while in the case of job types, employment contract, work schedule, and weekly work hours had significant correlation with presenteeism. Furthermore, the analysis of psychosocial factors’ association with presenteeism showed that 15 of 18 items increased presenteeism with statistical significance.

Aronsson et al. [21] pointed out that groups of low-income earners and people with financial problems show higher rates of presenteeism than their counterparts. Similarly, this study found that presenteeism is higher in those with an income level of “less than 2 million” than it is in those with an income level of “greater than 2 million.” That is, people in financially difficult situations attend work despite being sick. Aronsson et al. [2] also pointed out that presenteeism increases when it is difficult to find substitute workers, and Caverley et al. [22] noted in their study of Canadian civil servants the deficiency in backup human resources resulting from downsizing as the major cause for presenteeism. These study results reflect that presenteeism is higher among full-time workers than among part-time workers and higher in those with regular work shifts and greater weekly work hours. These factors are assumed to be evidence of people being forced to attend work because people under these conditions are responsible for completing tasks that only they can undertake.

Regarding ostracizing and bullying harassment within workplaces, Lu et al. [28] have derived significant values of exposure to physical violence and threats of violence within workplaces as factors affecting absenteeism while Hoel et al. [32] report presenteeism, stress, and social cost loss resulting from bullying harassment. In this study, all factors regarding bullying harassment within workplaces except for the ostracizing factor showed statistically significant positive association with presenteeism (age, education, regional and sexual discrimination, employment contract, physical violence, and sexual harassment). This means that the workers’ mental and physical harm resulting from the aforementioned factors exist unexposed and can cause problems in individual health as well as in the social-cost and social-harmony aspects resulting from decreases in productivity. In particular, the fact that sexual harassment shows the highest odds ratio (2.90, CI 2.01–4.19) among the related factors indicates the relatively weak women’s status within workplaces.

The reason for job insecurity increasing the presenteeism risks is assumed to be associated with attending work while ill in fear of losing work [24]. This situation is evidently applied to the temporary workers [33] and suggests that many workers in domestic temporary positions are at high presenteeism risk. Temporary position workers were not analyzed in this study; future studies are needed for the matter.

Poor work-life balance also significantly increased presenteeism risks, which is similar to previous study results [34, 35]. It is one of the 10 items specified by EU-OSHA (2007) as psychosocial risk factors, and resultant job stress has been noted recently [36].

Many studies have been conducted on the association between presenteeism and stress both domestically and internationally. Boles et al. [26] mention that stress adversely affects presenteeism, and Ryu et al. [17] and Jung et al. [18] have analyzed stress by categorizing it into job stress and psychosocial stress as presenteeism factors. In looking at the results of this study, presenteeism increases in proportion with lower levels of job satisfaction and colleague support; higher levels of job stress, work intensity, and job strain; and more emotional labor. This indicates that stress greatly affects workers’ health and productivity [37]. Job autonomy and presenteeism did not show statistically significant association, which corresponds with the study by Johansson et al. [5], presumably because workers with high job autonomy do not realize that they are ill or take rest or vacation instead of attending work while ill.

The limitations of this study can be described as follows. First, being a cross-sectional study through work environment surveys, this study had limitations in investigating causality between presenteeism and psychosocial factors within workplaces. Thus, progressive studies are needed to investigate their association. Second, only wage earners were analyzed in investigating the association between presenteeism and various psychosocial factors at diverse workplaces, thus making the results difficult to apply to other groups. Third, the investigation of the psychosocial factors was limited to several questionnaire items rather than using proven scales for each. However, there are not yet many proven scales available in Korea. Additionally, considering the study by Wanous et al. [38], in which one question had enough credibility for evaluating job satisfaction in comparison with the scales composed of various items, other items are considered to be trusted to some degree. Lastly, by defining presenteeism as “attending work while ill,” the study is limited in its ability to reflect various other definitions of presenteeism. However, it is considered that presenteeism can be sufficiently explained with the definition of presenteeism used in this study since this study has been conducted based on the surveyed data with prescribed statements and questions, and there are considerable similarities among the various definitions.

The merits of this study are as follows. First, the sample was large enough to represent the wage earners in Korea since secondary data with a large population were used. Second, interpretation and selection biases were minimized because trained interviewers handled the surveys using the structured questionnaires. Third, various psychosocial factors within workplaces that were not included in the previous studies were used, which can draw various study methods related to presenteeism in the future. This study confirmed that there is significant correlation between presenteeism and psychosocial factors within workplaces, and this means that presenteeism results from various factors involved rather than simply from individual health problems. To decrease presenteeism, interests and efforts are needed both individually and socially, systematic measurement methods must be established for psychosocial factors within workplaces, and more in-depth studies are needed on their association with presenteeism in the future.

## Conclusion

This study investigated the association between presenteeism and various psychosocial factors within workplaces with wage earners as participants based on data from the third KWCS. As with previous study, this study investigated the factors considered to be related to absenteeism, and their associations with presenteeism were confirmed [23].

EU-OSHA (2007) specified psychosocial factors within workplaces, job-related stress, violence, discrimination, bullying, and harassment as new major issues that threaten job health and safety [36]. The association of these factors with presenteeism has been confirmed in this study as well. These factors are often found in socially vulnerable people, i.e., younger or handicapped people, women, foreigners, and temporary position workers. Thus, legal and systematic safety networks for socially vulnerable people must be well maintained.

Work–life balance is also a noted concept of late. There have been discussions and studies on individual living needs and their harmony with work situations as the 5-day workweek system was implemented in Korea. Additional studies are needed to investigate the more precise association between presenteeism and work–life balance.

Various psychosocial factors within workplaces used in this study fall in the subareas under the Korean Occupational Stress Scale [37], which must be considered in future studies investigating the more precise association between presenteeism and various related factors using proven scales. Additionally, since presenteeism greatly affects the mental and physical health of individuals, enterprise productivity, and social cost, concrete discussions are needed on various measures to decrease presenteeism.
